# The role of brain derived neurotrophic factor in central nervous system

**DOI:** 10.3389/fnagi.2022.986443

**Published:** 2022-09-08

**Authors:** Yiyi Li, Fang Li, Dongdong Qin, Hongyu Chen, Jianhao Wang, Jiabei Wang, Shafei Song, Chao Wang, Yamei Wang, Songyan Liu, Dandan Gao, Zhi-Hao Wang

**Affiliations:** ^1^Department of Neurology, Renmin Hospital of Wuhan University, Wuhan, China; ^2^Center for Neurodegenerative Disease Research, Renmin Hospital of Wuhan University, Wuhan, China

**Keywords:** brain derived neurotrophic factor, physiological, pathological, treatment, central nervous system

## Abstract

Brain derived neurotrophic factor (BDNF) has multiple biological functions which are mediated by the activation of two receptors, tropomyosin receptor kinase B (TrkB) receptor and the p75 neurotrophin receptor, involving in physiological and pathological processes throughout life. The diverse presence and activity of BDNF indicate its potential role in the pathogenesis, progression and treatment of both neurological and psychiatric disorders. This review is to provide a comprehensive assessment of the current knowledge and future directions in BDNF-associated research in the central nervous system (CNS), with an emphasis on the physiological and pathological functions of BDNF as well as its potential treatment effects in CNS diseases, including depression, Alzheimer’s disease, Parkinson’s disease, Huntington’s disease, amyotrophic lateral sclerosis, multiple sclerosis, and cerebral ischemic stroke.

## Introduction

Brain derived neurotrophic factor, a member of the neurotrophin family, plays a critical role not only in the growth and development of the nervous system but also in supporting neurons survival and facilitating neurogenesis. In addition, it participates in glutamatergic and gamma-aminobutyric acid (GABA)-ergic synaptic plasticity and affects serotonergic and dopaminergic neurotransmission (Colucci-D’Amato et al., 2020). It is widely distributed in the cortical region, hippocampus, and visual cortex, as well as other parts of CNS such as substantia nigra, striatum, retrorubral region, and ventral tegmental area. The expression level of BDNF in brain tissues is determined by transcription of BDNF mRNA and translation of BDNF protein.

Brain derived neurotrophic factor signals are mediated by TrkB receptor and p75 neurotrophin receptor. The former is the key receptor for BDNF in adult brain since its wide range of expression and a higher binding affinity for BDNF than the latter. TrkB receptor has tyrosine residues in its kinase domain, and phosphorylated-TrkB triggers downstream signaling cascades such as phospholipase C (PLC), mitogen-activated protein kinase (MAPK) and phosphatidylinositol 3-kinase (PI3K), exerting neuronal protective effects. However, truncated TrkB without intracellular kinase domain possesses inhibitory effect against mature BDNF signaling which activates full length TrkB. Furthermore, pro-BDNF is preferential to bind with p75 neurotrophin receptor, which has a low affinity to mature BDNF and leads to negative effects such as neuronal cell death (Teng and Hempstead, 2004).

It was reported that BDNF is highly active in cortex, hippocampus, and basal forebrain (Mirowska-Guzel et al., 2013). The diverse presence of BDNF leads to its important involvements in multiple neurological and psychiatric disorders. In this review, we briefly summarize the current state of knowledge regarding the physiological and pathological functions of BDNF as well as its potential treatment effects in CNS diseases.

## Physiological functions of brain derived neurotrophic factor

Brain derived neurotrophic factor is mainly produced by neurons and glial cells and serves as a pleiotropic role in CNS (Lessmann and Brigadski, 2009). It is essential for neuronal genesis, differentiation, survival and growth, and acts as a mediator, modulator, or instructor of synaptic plasticity, viability and transmission (Kowiański et al., 2018). There are two forms of BDNF in human brain, the BDNF precursor (proBDNF) and mature BDNF (mBDNF). The proBDNF stored in either dendrites or axons (Lessmann et al., 2003), is synthesized as pre-proBDNF in endoplasmic reticulum followed by removing signal peptide in golgi apparatus to form proBDNF eventually (Wang M. et al., 2021). The mBDNF is converted from proBDNF which undergoes cleavage and strips off the pro-domain (BDNF pro-peptide, pBDNF) intracellularly (by furin or PC1/3/7) or extracellularly (by tPA/plasmin system or matrix metallopeptidases) (Lee et al., 2001; Mowla et al., 2001; Wang M. et al., 2021).

Brain derived neurotrophic factor was released both by presynaptic and postsynaptic terminals (Matsuda et al., 2009) as a mixture of pro- and mature forms in an activity dependent manner (Pang et al., 2004). Two types of BDNF maintain a dynamic balance and the ratio between them varies at different stages of brain development. During early postnatal period, proBDNF reaches a high concentration, while mBDNF is more dominant in adulthood (Yang et al., 2009, 2014). mBDNF takes effect by binding with two kinds of plasma membrane receptors, the TrkB receptor (Martin-Zanca et al., 1986) and the p75 neurotrophin receptor (Dechant and Barde, 2002). mBDNF, as a member of the neurotrophin family, has a high affinity (dissociation constant ∼10^11^ M) to TrkB receptor (Rodriguez-Tébar and Barde, 1988), which promotes cell survival (Volosin et al., 2006), facilitates long term potentiation (LTP) and increases spine complexity (Zagrebelsky et al., 2005). However, mBDNF has a low affinity (dissociation constant ∼10^9^ M) to p75 neurotrophin receptor (Meeker and Williams, 2015), which preferentially binds to proBDNF, and the latter signal involves in brain development, facilitates long term depression (LTD) and induces apoptosis (Woo et al., 2005; Friedman, 2010). Due to its critical role in LTP, BDNF has been assumed to be an essential part of supporting memory formation and maintenance by promoting synaptic integration (Bramham and Messaoudi, 2005). Furthermore, BDNF increases neurogenesis by promoting cell survival and proliferation (Katoh-Semba et al., 2002; Lee S. H. et al., 2007).

**FIGURE 1:**
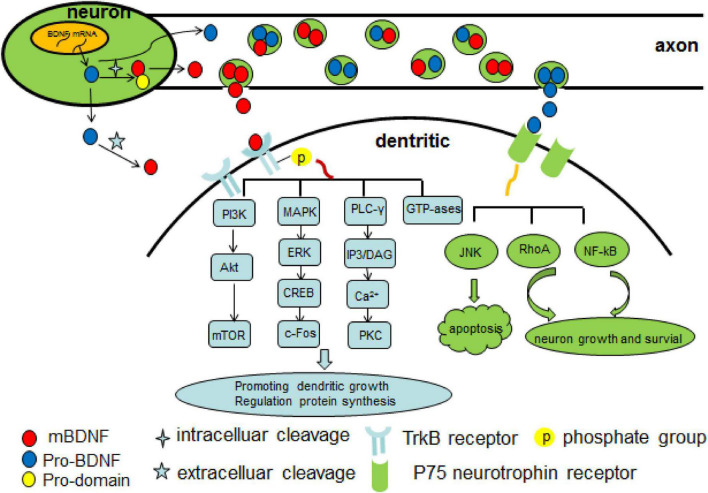
Overview of Brain derived neurotrophic factor (BDNF) signaling cascade. There are two forms of BDNF–proBDNF and mBDNF. The proBDNF is initially synthesized and then cleaved intra or extracellularly into mBDNF. Two types of BDNF activate several important intracellular downstream signaling cascades as above shown by mediating TrkB receptor and p75 neurotrophin receptor. Pro-domain BNDF propeptide, PI3K phosphatidylinositol 3-kinase, MAPK mitogen-activated protein kinase, ERK extracellular-signal-regulated kinase, CREB Camp response element-binding protein, c-Fos transcription factor c-Fos, PLC-γ phospholipase C-γ, IP3 inositol 1,4,5-trisphosphate, DAG 1,2-diacylglycerol, PKC protein kinase C, GTP-ases guanosine triphosphate hydrolases, JNK c-Jun amino terminal kinase, RhoA Ras homolog gene family member, NF-kB nuclear factor kappa B.

As is mentioned above, BDNF takes actions mainly by binding to TrkB, which is abundantly expressed in hippocampal neurons. After binding, the BDNF/TrkB complex is then internalized into the neuron and serves as a docking site for diverse signaling cascades, protein phosphorylation cascades, and secondary signaling systems (Huang and Reichardt, 2003; Nykjaer et al., 2005; Yoshii and Constantine-Paton, 2010). The binding of BDNF to TrkB receptor leads to phosphorylation of TrkB, thus activating several important intracellular downstream signaling cascades, including PLC, phosphatidylinositol 3-kinase/protein kinase B (PI3K/AKT), mitogen-activated protein kinase/extracellular signal-related kinase (MAPK/ERK) and guanosine triphosphate hydrolases (GTP-ases) of the Ras homolog (Rho) gene family pathways (Minichiello, 2009; Mohammadi et al., 2018). The BDNF/TrkB/PKC signaling is essential for synaptic function and maintenance (Lanuza et al., 2019), and it links pre- and post-synaptic activity to maintain neuromuscular function, which can be affected by a decrease in neuromuscular activity such as occurring neuromuscular disorders (Hurtado et al., 2017). BDNF/TrkB/PI3K/Akt pathway has an antiapoptotic effect and suppresses autophagy by which further inhibits the degradation of three important postsynaptic proteins (PSD-95, PICK1, and SHANK3) that is essential for N-methyl-D-aspartate receptor (NMDAR)-dependent synaptic plasticity (Nikoletopoulou et al., 2017). Synaptic plasticity is a dynamic process that involves in competition between Ca2+/calmodulin-dependent protein kinase II (CaMKII) and PSD-95 for binding to the NR2A NMDAR subunit, while LTP is associated with an increase of αCaMKII binding with the NR2A/B subunit of the NMDA receptor and a concomitant decrease binding with PSD-95 (Stachowicz, 2022). In addition, BDNF also has direct effect on NMDA receptor subunits but with different level. Acute stimulation of hippocampal neurons with BDNF rapidly upregulated the protein levels and delivery to the plasma membrane of the NR1 and NR2B NMDA receptor subunits, while with a delayed increase of NR2A subunit (Caldeira et al., 2007). Another study revealed that BDNF increased Girdin phosphorylation through the TrkB/PI3K/Akt pathway leading to phosphorylation of the NR2B subunit and NMDA receptor activation, which is crucial for synaptic plasticity and memory (Nakai et al., 2014). The PI3K/Akt/mTOR pathway promotes dendritic growing and branching through regulation of protein synthesis and cytoskeleton development (Jaworski et al., 2005; Kumar et al., 2005). The MAPK/ERK pathway is pivotal not only for early response gene expression such as c-Fos but also for cytoskeleton protein synthesis such as Arc and cypin (Gonzalez et al., 2016). Moreover, it plays a critical role in dendritic growth and branching of hippocampal neurons (Kwon et al., 2011). In addition, through BDNF/AMPAR/mTOR signaling, ketamine induces structural plasticity in mouse mesencephalic and human induced pluripotent stem cells-derived dopaminergic neurons (Cavalleri et al., 2018). The BDNF/TrkB/GTP-ases pathway promotes actin and microtubule synthesis, resulting in growth of neuronal fibers (Gonzalez et al., 2016). The pro-BDNF/p75 neurotrophin receptor/sortilin binding complex initiates signaling cascades leading to activation of c-Jun amino terminal kinase (JNK), ras homolog gene family member A (RhoA), and nuclear factor kappa B (NF-kB) downstream, which involves in neuronal apoptosis (Anastasia et al., 2013), neuronal growth cone development and motility (Meeker and Williams, 2015), and neuronal survival (Lee et al., 2001; Figure 1).

## Pathological functions of brain derived neurotrophic factor

**FIGURE 2:**
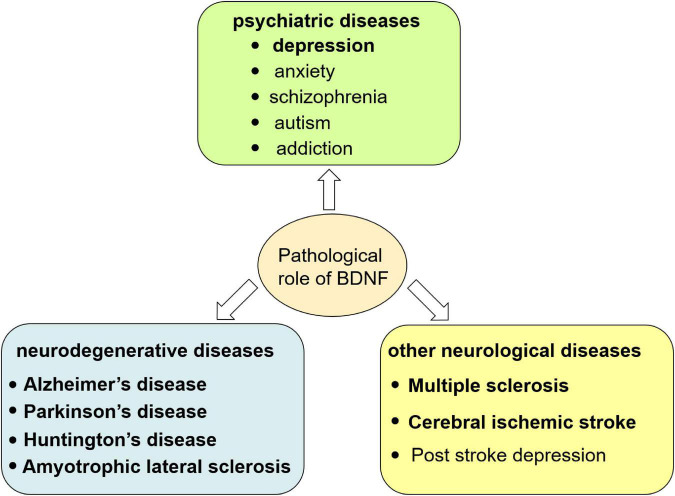
Pathological role of BDNF in central nervous system. The dysfunction of BDNF signaling causes neurological disorders including psychiatric, neurodegenerative diseases and other neurological diseases.

The change of BDNF level in both peripheral blood and CNS and the imbalance or insufficient of pro-BDNF transformation into mBDNF have been found to be involved in the pathogenesis of multiple diseases such as depression, Alzheimer’s disease, Parkinson’s disease, Huntington’s disease, amyotrophic lateral sclerosis, multiple sclerosis, and cerebral ischemic stroke (Eyileten et al., 2021; Wang M. et al., 2021). In these diseases, failure of the switch of pro-BDNF to mBDNF is due to the abnormal proteolytic cleavage. For example, altered expression and/or activities of components in the tPA/plasmin system (extracellular cleavage proteases) participated in pathological processes related to depression and anxiety (Wang M. et al., 2021). Details will be summarized as below (Figure 2 and Table 1) and introduced in the following seven sections.

**TABLE 1:** Brain derived neurotrophic factor (BDNF) level in different brain disorders and BDNF associated therapy.

Disorders	BDNF level in patients and animal model	Therapy	References
Depression	↓BDNF in hippocampus and prefrontal cortex ↓BDNF in serum and plasma ↑Serum proBDNF ↓serum mBDNF ↓pBDNF in human cerebrospinal fluid ↑pBDNF and proBDNF ↓mBDNF in parietal cortex	antidepressant drugs electroconvulsive therapy peripheral injection of BDNF anti-proBDNF antibody 7,8-DHF	Nibuya et al., 1995; Duman and Monteggia, 2006; Kim et al., 2007; Lee B. H. et al., 2007; Schmidt and Duman, 2010; Zhou et al., 2013; Bai et al., 2016; Zhang et al., 2016; Jiang et al., 2017; Yang et al., 2017; Kojima et al., 2019
Alzheimer’s disease	↓serum BDNF ↑proBDNF and proBDNF/BDNF in human cerebrospinal fluid ↑pBDNF and pBDNF/BDNF in hippocampal specimens Aβ impeded BDNF/TrkB downstream signaling Tau inhibited BDNF production	7,8-DHF CF3CN	Lim et al., 2015; Rosa et al., 2016; Aytan et al., 2018; Fleitas et al., 2018; Ng et al., 2019; Chen et al., 2021
Parkinson’s disease	↓serum BDNF ↓BDNF in both brain and gut ↓Serum mBDNF and mBDNF/proBDNF	Dopamine neuron protective drugs intracerebral injection of BDNF non-invasive vagus nerve stimulation deep brain stimulation 7,8-DHF	Moreau and Destée, 2009; Chen et al., 2017; Ahn et al., 2021; Faust et al., 2021; Mondal et al., 2021; Yi et al., 2021; Tian et al., 2022
Huntington’s disease	↓BDNF mRNA and protein levels in the cortex	BDNF overexpressing human neural stem cells transplantation bone-marrow mesenchymal stem cells transplantation P42 and BDNF combination treatment 7,8-DHF	Zuccato et al., 2008; Dey et al., 2010; García-Díaz Barriga et al., 2017; Kim et al., 2020; Couly et al., 2021
Amyotrophiclateral sclerosis	↓BDNF protein level	stem cell therapy 7,8-DHF	Korkmaz et al., 2014; Schiaffino et al., 2018; Forostyak et al., 2020; Wang J. et al., 2021
Multiple sclerosis	↓BDNF protein level	stem cells transplantation 7,8-DHF	Makar et al., 2016; Naegelin et al., 2020; Brown et al., 2021
Cerebral ischemic stroke	↓BDNF in MCAO mice hippocampal ↓serum BNDF in post stroke depression patients ↓BDNF at the acute phase of CIS	stem cells therapy neuron protective drugs BDNF treatment	Nagahara and Tuszynski, 2011; Lasek-Bal et al., 2015; Cook et al., 2017; Kaur et al., 2021

### Brain derived neurotrophic factor and depression

Depression is not an unusual psychiatric disease at present. Major depressive disorder is characterized by emotional, motivational, cognitive and physiological domain symptoms, making it a complicated disease to treat. BDNF/TrkB signaling is involved in various psychiatric diseases and is widely studied in the context of depression, anxiety disorders, schizophrenia, autism, and addiction (Autry and Monteggia, 2012). Here, we mainly discuss the role of BDNF in depression. Clinical studies have shown that patients with severe depression have a low level of BDNF in the hippocampus and prefrontal cortex, accompanied by cerebral hippocampus atrophy, neuronal apoptosis and synaptic loss (Duman and Monteggia, 2006). In addition, the serum BDNF level was also found to be decreased in depression patients (Lee B. H. et al., 2007), and the level of plasma BDNF seems to be lower in patients with severe depression compared to mild depression patients (Kim et al., 2007). However, treatment with conventional antidepressant drugs as well as electroconvulsive therapy enhanced the mRNA expressions of BDNF and TrkB in the hippocampal and cortical regions (Nibuya et al., 1995). Several intracellular signaling pathways such as mammalian target of rapamycin (mTOR) and glycogen synthase kinase 3 (GSK-3) have been implicated in the antidepressant effects of ketamine (Li et al., 2010; Beurel et al., 2011). Whereas, previous works examining the antidepressant effects of ketamine demonstrated that mTOR was a downstream target of BDNF (Autry et al., 2011). Animal model of depression revealed that BDNF signaling was rescued in the hippocampus after peripheral injection of BDNF for 14 consecutive days, and its mRNA level in the CA3 area of the hippocampus was also increased, suggesting the antidepressant-effect of BDNF (Schmidt and Duman, 2010). All these mentioned indicate that up-regulating BDNF level may have positive effect to the treatment of depression. Some clinical studies revealed that the serum proBDNF level in severe depression patients was apparently higher than that in healthy subjects, while the level of mBDNF shows the opposite trend. What’s more, the ratio of mBDNF/proBDNF was much lower in depression patients compare to healthy control, while was increased after antidepressant drugs treatment (Zhou et al., 2013; Jiang et al., 2017). Another study showed that, in human cerebrospinal fluid, levels of pro-peptide BDNF (pro-domain of BDNF, pBDNF) were significantly lower in patients with major depressive disorder than in controls (Kojima et al., 2019). In addition, autopsy results of depression patients showed that the expressions of pBDNF and proBDNF increased in parietal cortex while mBDNF decreased (Yang et al., 2017). Furthermore, intraperitoneal or intraventricular injection of anti-proBDNF antibody can neutralize proBDNF and alleviate depression-like behavior in rats (Bai et al., 2016). A research demonstrated that chronic 7,8-DHF (7,8-Dihydroxyflavone, a TrkB agonist) treatment rescued the depressive-like behaviors in sucrose preference test and novelty suppressed feeding test by regulating TrkB signaling, increasing BDNF levels and promoting synaptic protein expression (Zhang et al., 2016). In summary, overall consideration of the dynamic equilibrium and mutual interaction between proBDNF and mBDNF as well as pBDNF is of more significant than merely focusing on mBDNF.

### Brain derived neurotrophic factor and Alzheimer’s disease

Alzheimer’s disease (AD) is the most common form of dementia, which was pathologically characterized by extracellular senile plaque (SP) formed by the deposition of β-amyloid (Aβ), and intracellular neurofibrillary tangles (NFTs) formed by the abnormally phosphorylated Tau protein aggregation as well as vascular amyloidosis and loss of neurons in the cortex and hippocampus (Edler et al., 2017; Szaruga et al., 2017). Multiple studies showed that BDNF, proBDNF and pBDNF played important roles in the occurrence and development of AD. A meta-analysis indicated that AD patients, rather than mild cognitive impairment patients, have significantly lower serum BDNF levels compared to age-matched healthy controls, and a significant decrease in peripheral BDNF levels can only be detected at the late stage of dementia (Ng et al., 2019). A study on AD patients showed a negative correlation between cognitive decline and BDNF mRNA level in the dorsal lateral prefrontal cortex (Buchman et al., 2016). Furthermore, significant increases in proBDNF expression and the ratio of proBDNF/BDNF in AD patients’ cerebrospinal fluid were detected, which led to an increase of pathogenicity and a decrease of trophic effect and caused neuronal apoptosis through p75 neurotrophin receptor (Fleitas et al., 2018). In hippocampal specimens of AD patients, the level of pBDNF was 16 times higher than that in healthy individuals, and the ratio of pBDNF/BDNF was 30 times higher compared to control. What’s more, cell death rate increased when treating cells with pBDNF and Aβ simultaneously compared to giving pBDNF solely, which indicated that they may cause synergistic toxicity in AD (Lim et al., 2015). In addition, Aβ promoted the production of shortened TrkB receptor mRNA and the decomposition of the full-length TrkB receptor, which impeded BDNF/TrkB downstream signaling (Jerónimo-Santos et al., 2015). Interestingly, we have found that deficiency in BDNF/TrkB neurotrophic signaling can increase CCAAT/enhancer binding protein β (C/EBPβ) expression and then activate δ-secretase which leads to APP and Tau fragmentation and causes AD eventually (Wang et al., 2019). On the other hand, excessive or pathologically altered Tau can inhibit BDNF production and neurotoxicity without Tau gene mutation or neurofibrillary tangles formation (Rosa et al., 2016). Reversely, BDNF can also impede phosphorylation of Tau (Jiao et al., 2016).

In all, the interactions between BDNF and Aβ or Tau need further investigation to find an effective therapy for AD. In 5xFAD mice models, researchers found that 2 months’ treatment of 7,8-DHF decreased cortical Aβ plaque deposition and protected dendritic arbor complexity in cortical neurons but without huge impact on the density of dendritic spines (Aytan et al., 2018). In addition, CF3CN, an optimized synthetic 7,8-DHF chemical, has the ability to activate TrkB neurotrophic signaling and inhibits the activation of delta-secretase, which attenuates AD pathologies and alleviates cognitive dysfunctions in 5xFAD mice. All these suggest that 7,8-DHF or CF3CN may be potential therapeutic agents for the treatment of AD (Chen et al., 2021).

### Brain derived neurotrophic factor and Parkinson’s disease

Parkinson’s disease (PD), the second most prevalent neurodegenerative disease in the world, is characterized by progressive degeneration of nigrostriatal dopaminergic neurons and the development of intracellular proteinaceous aggregates. It is a combination of motor and non-motor symptoms. The former which weights most contains tremor, bradykinesia, rigidity, and postural instability, and the latter involves in hyposmia, sleep disorders, autonomic nervous dysfunction, and mental disorders. Study shows that the non-motor symptoms not only precede but often are accompanied by PD, of which neuropsychiatric symptoms including depression, anxiety, apathy, hallucinations and impulse control disorders are up to 60% in PD patients, in which almost 20-40% suffer from depression (Song et al., 2015). Many studies have shown that the mRNA and protein levels of serum BDNF in patients with depression and PD were significantly reduced. What’s more, BDNF gene polymorphism, especially the Met allele, is associated with a higher neuropsychiatric burden in PD (Ramezani et al., 2020). An interesting clinical study has found that PD patients with higher self-rating Depression Scale (SDS) score show lower serum BDNF level (Chen et al., 2017). On the other hand, the decrease of BDNF in PD patients may also affect neuronal function in hippocampus, prefrontal cortex and amygdala and eventually leads to depressive symptoms (Enomoto et al., 2016). Therefore, some researchers recognized serum BDNF as a clinical biomarker for motor severity in depressed PD patients, especially in female (Huang et al., 2021).

Except for these neuropsychiatric symptoms, BDNF also takes effect in motor symptoms and other non-motor symptoms. BDNF supports the midbrain dopaminergic neurons surviving, promotes differentiation, and protects dopamine neurons from neurotoxins (Minichiello et al., 2002). BDNF overexpression alleviated motor deficits and cognitive impairment in MPTP-induced PD mice through mitigating mitochondrial damage (Chang and Wang, 2021). What’s more, BDNF level was significantly decreased in the colon of MPTP-treated group compared to the vehicle-treated group, indicating a role of BDNF in the gut-brain axis in PD (Choi et al., 2021). In accordance with this, BDNF and Netrin-1 are strongly decreased in both brain and gut of PD patients, and conditionally knocking out of these trophic factors in gut leads to dopaminergic neuronal loss, constipation and motor deficits (Ahn et al., 2021). A clinical study included one hundred and fifty-six patients with limb tremor and/or bradykinesia who meet the MDS Parkinson’s diagnostic criteria and analyzed their serum level of proBDNF and mBDNF. They found that serum levels of mBDNF and mBDNF/proBDNF were significantly lower in the PD group compared with non-PD group, while the proBDNF showed opposite result. Thus, they supposed that mBDNF/proBDNF can be used as biomarker for early stage of PD and the combination of mBDNF and proBDNF has better diagnostic value than mBDNF alone in PD diagnosis (Yi et al., 2021).

Over the past decades, most of drugs for PD treatment mainly focus on modulating dopamine concentrations in the basal ganglia. Recently, studies of neurotrophic factors such as glial cell line-derived neurotrophic growth factor and BDNF, which are considered the primary factors for neuroprotection in PD, have gained a great attention. Intracerebral injection of BDNF markedly improves levodopa-induced dyskinesia in PD rat model without affecting the therapeutic effect of levodopa (Moreau and Destée, 2009). BDNF is being developed as a neuron protective drug, and the underlying mechanisms − include promoting neuronal regeneration, repairing the damaged neurons, promoting functional recovery, and enhancing the activity of antioxidant enzymes. However, exogenous BDNF cannot cross the blood-brain barrier, therefore, the treatment of PD is still under research. A recent study has shown that non-invasive vagus nerve stimulation, which is an established neurostimulation therapy used in the treatment of epilepsy, migraine and cluster headache, is demonstrated to improve gait and motor function in PD patients, and it can significantly increase BDNF level (Mondal et al., 2021). What’s more, the level of BDNF in the motor cortex increased after deep brain stimulation (DBS) in the subthalamic nucleus (STN), which is a powerful therapeutic alternative for the treatment of PD (Faust et al., 2021).

The α-Synuclein (α-Syn) is a major component of Lewy body which is a pathological marker of PD. Physiologically, α-Syn takes effect in intercellular dopamine storage, synaptic membrane biogenesis, and lipid transport (Lotharius and Brundin, 2002). Wild-type α-Syn induces BDNF expression, while α-Syn mutant (A30P and A53T) cannot induce BDNF expression (Kohno et al., 2004). These α-Syn mutants tend to form protofibrils and further aggregate into larger inclusion bodies associated with PD, which significantly inhibit axonal transport of BDNF/TrkB (Papapetropoulos and Mash, 2005; Volpicelli-Daley et al., 2014). However, BDNF reduces the degradation of TrkB through inhibiting the formation of the α-Syn –TrkB complex (Kang et al., 2017). Further studies are needed to clarify the crosstalk between BDNF/TrkB and the biomarkers of PD such as α-Syn and find effective treatment for neuronal degeneration. Recently, a study found that 7,8-DHF could ameliorate α-Syn 1-103-induced mitochondrial impairment and motor dysfunction, indicating a novel oral bioactive therapeutic agent for the treatment of PD (Tian et al., 2022).

All in all, BDNF plays an important role in the diagnosis and the treatment of PD, as well as involving in the development of PD through affecting PD pathology.

### Brain derived neurotrophic factor and Huntington’s disease

Huntington’s disease (HD) is an autosomal dominant neurodegenerative disease caused by an expanded CAG repeat sequence in the huntingtin gene which leads to an abnormal expansion of the polyglutamine tract in the N-terminus of the protein huntingtin. This mutant huntingtin protein misfolds, aggregates, and disrupts proteostasis, which leads to degeneration of striatal medium spiny neurons (Rosas et al., 2003). Clinically, it is a progressive motor, cognitive, and mental dysfunction disease, whose mean onset age is 35 to 44 years and median survival time is 15 to 18 years once onset (Caron et al., 1993), and unfortunately, it affects the quality of life of their offspring and their partners.

The relationship between serum (or plasma) and cerebrospinal fluid levels of BDNF and HD has been controversial. A recent report concluded that levels of BDNF in plasma and cerebrospinal fluid are not a biomarker for HD (Ou et al., 2021). It is reported that the striatum cannot produce BDNF, and it maintains its function depending on BDNF from the cortex (about 95%), thalamus and mesencephalon (Bawari et al., 2019). BDNF, which is synthesized in cortical neurons and then delivered into striatum by anterograde transportation, is essential for the cortico-striatal synaptic activity and the survival of GABAergic neurons. Depletion of BDNF in the cortex of HD patients may make striatal neurons more vulnerable to injury (Zuccato et al., 2001). A study showed significant reduction of BDNF mRNA and protein levels in the cortex of HD patients through a systematic and quantitative assessment (Zuccato et al., 2008). Additionally, it is found that the delivery of BDNF is attenuated while transferring from the cortex to the striatum in an animal model of HD. Therefore, the reduction of cortical BDNF delivery would result in decreased activity of the cortex and striatum synaptic activity and synaptic loss (Yu et al., 2018). BDNF pro-domain knockout mouse showed impaired righting reflex, abnormal motor behaviors, obvious weight loss and short lifespan, which displayed a Huntington’s disease-like phenotype, supporting the BDNF hypothesis in the pathogenesis of HD (Li et al., 2020). Furthermore, low expression of BDNF in HD pathogenesis is potentially mediated by cAMP, MAPK and Ras signaling pathways (Zhou et al., 2021).

The astrocyte constitutes the largest population of brain cells and plays multiple roles, one of which is that it involves in neuronal dysfunction of HD. Expression mutant huntingtin with 160 polyQ specifically in astrocytes causes age-dependent neurological symptoms in transgenic mice, indicating a role of mutant huntingtin in exacerbating HD neuropathology (Bradford et al., 2009). What’s more, the mutation of huntingtin gene affects the processing and secretion of the BDNF in astrocytes, leads to progressive degeneration of striatal GABAergic medium spiny neurons and decreases BDNF level in the brain of HD patients (Wang et al., 2012; Bawari et al., 2019). In return, BDNF modulates striatal astrocyte function by inducing astrocyte to secrete soluble neuroprotective factors that selectively protect neurons expressing mutant huntingtin (Saba et al., 2020).

The potential therapeutic effects of BDNF for HD have been reported in various rodent models. In a rat model of HD, researchers transplanted BDNF-overexpressing human neural stem cells (HB1.F3.BDNF) into the contra-lateral side of unilateral quinolinic acid-lesioned striatum, and found that BDNF can promote migration, differentiation and functional restoration of HD rat (Kim et al., 2020). Another research showed that transplantation bone-marrow mesenchymal stem cells with BDNF over-expressed into striatum slowed neurodegenerative processes and alleviated behavioral disorder in the YAC 128 mouse model of HD (Dey et al., 2010). However, the long-term safety and efficacy of this approach still need further research. A dual therapy that combines P42 treatment which increases TrkB expression in striatum, with BDNF-enhancing therapy such as environmental enrichment efficiently delayed HD pathology in R6/2 mice (Couly et al., 2021). Furthermore, bilateral expression of BDNF by adeno-associated virus1/2 in the striatum alleviated motor and cognitive dysfunction in transgenic HD rats, accompanied by increased volume of the striatum and number of neural cells (Connor et al., 2016). In a more practical way, researchers found that oral 7,8-DHF ameliorated cognitive and motor deficits in a mouse model of HD through specific activation of the PLCγ1 pathway (García-Díaz Barriga et al., 2017). These results suggest that BDNF has a compensating effect to HD and might be a promising treatment for HD.

### Brain derived neurotrophic factor and amyotrophic lateral sclerosis

Amyotrophic lateral sclerosis (ALS) is an adult-onset neurodegenerative disorder, characterized by the loss and degeneration of both lower and upper motor neurons. The etiopathogenesis of ALS is still unknown, but the possible hypotheses include oxidative stress, inflammation, protein aggregation and miss-folding, as well as glutamate excitotoxicity, RNA processing and epigenetic dysregulation (Jankovic et al., 2021). Therefore, the treatments of ALS are of multi-level but with limited therapeutic effects.

As a member of the neurotrophin family, BDNF promotes cell regeneration and survival. A study conducted on spinal cord tissue in SOD1 G93A mice (a model of ALS) showed that the expression level of BDNF decreased significantly compared to control mice (Schiaffino et al., 2018). In addition, another study showed that physical exercise such as running and swimming can improve the BDNF/TrkB neurotrophic signaling at the neuromuscular junction and reduce the impact of ALS in mice (Just-Borràs et al., 2020). Based on the above evidence, recent studies have recognized the critical role of BDNF in maintaining motoneurons survival, and take BDNF as a potential treatment for ALS. BDNF have been used in ALS human clinical trials, but the results are disappointing due to the poor pharmacokinetics and pharmacodynamics of BDNF. Deletion of the BDNF receptor TrkB.T1 alleviated muscle weakness and motoneuron cell death of spinal cord in the G93A SOD1 animal model of ALS, indicating TrkB.T1 may limit the neuroprotective BDNF signaling to motoneurons via a non-cell autonomous mechanism, which providing new understanding of the reasons for past clinical failures (Yanpallewar et al., 2021). In a transgenic rat model, intraspinal transplantation of pluripotent stem cells might rescue or replace dying motoneurons and these cells display neuroprotective effects by regulating local gene expression and extracellular matrix plasticity of central nervous system, which means that stem cell therapy is a promising therapeutic strategy for ALS (Forostyak et al., 2020). Additionally, transplantation of BDNF-overexpressing human umbilical cord mesenchymal stem cell-derived motor neurons was capable of increasing motor ability and prolonging lifespan of hSOD1 G93A mice (Wang J. et al., 2021). What’s more, chronic administration of 7,8-DHF significantly improved motor deficits, and preserved spinal motor neurons count and dendritic spines in SOD1 G93A mice (Korkmaz et al., 2014). In summary, BDNF and its equivalent drugs may be a promising therapeutic strategy for ALS, but further researches are needed to overcome the difficulties of poor pharmacokinetics and pharmacodynamics of BDNF.

### Brain derived neurotrophic factor and multiple sclerosis

Multiple sclerosis (MS) is a chronic autoimmune disease characterized by neuronal inflammation, degeneration and demyelinating lesions within brain and spinal cord (primarily in the white matter of the brain), which leads to motor dysfunction and cognitive decline. The mechanisms underlying the pathogenesis and progression of MS are not fully understood and current treatments have limited efficacy. Several molecules such as BDNF, IL-1β, PDGF, and CB1Rs, are involved in functional recovery of MS from the acute phase and are thus taken as potential therapeutic targets (Ksiazek-Winiarek et al., 2015). Recently, a novel drug, 7,8-DHF was found to reduce the clinical and pathological severity of MS in a murine model through activation of TrkB/AKT/STAT3 signaling and reduction of inflammation and demyelination (Makar et al., 2016), which brought good news to this disease.

The versatile BDNF acts as a neuroprotective factor in the process of inflammation, degeneration and demyelination of MS. BDNF is required to drive the endogenous repair of regeneration and remyelination after demyelinating inflammatory injury in MS, which is crucial for neuronal preservation and prevention of clinical progression (Brod, 2022). Study showed that neural stem cells transplantation rescued the progression of MS by inducing anti-inflammation and promoting neurogenesis and myelination, possibly by modulating BDNF and FGF signaling pathways in an experimental autoimmune encephalomyelitis mouse model of MS (Brown et al., 2021). Another study revealed that BDNF is initially presented in T cells and macrophages in MS lesions to confirm the capability of producing BDNF in human immune system. However, high levels of cytokines tend to negatively regulate circulating BDNF levels indicating BNDF may act as an immune-mediated defense of neurons in MS lesions (Sorenson et al., 2014). To determine whether BDNF can serve as a biomarker for MS, a comparatively large cohort research found that the level of BDNF can be detected to have a difference between MS patients and healthy controls, with mean BDNF levels in the former were lower by 8% than the latter. However, the difference is minor and thus it is unlikely to effectively evaluate and inform decision-making processes at an individual patient level. Therefore, it is hard to claim that serum BDNF is a biomarker for MS although it has multifunction in this disease (Naegelin et al., 2020).

### Brain derived neurotrophic factor and cerebral ischemic stroke

Cerebral stroke is one of the leading causes of death and long-term disability worldwide, of which cerebral ischemic stroke (CIS) takes up the overwhelming majority. Recently, BDNF has been focused on in CIS, especially its relationship with post-stroke mobility. A study found that the expression of BDNF and TrkB decreased both in oxygen glucose deprivation cells and in mice hippocampal experiencing surgery of middle cerebral artery occlusion (MCAO). Shuxuening injection reverses it and promotes the recovery of post-stroke cognitive and motor deficiencies via BDNF-mediated Neurotrophin/Trk Signaling (Li et al., 2021). Furthermore, intra-arterially mesenchymal stem cells therapy in post-stroke facilitates neuroprotection and regulates ER stress-mediated apoptosis via the BDNF/TrkB signaling pathway (Kaur et al., 2021). Additionally, BDNF is also involved in post stroke depression (PSD). PSD patients show lower level of serum BNDF compared to those without PSD. Furthermore, antidepressants could improve the expression of BDNF in brain, which further alleviates depression symptoms. However, the mechanisms of BDNF in the development of PSD are still unknown (Zhang and Liao, 2020).

Except for post-stroke, it has been reported that BDNF level was significantly decreased at the acute phase of CIS and it can act as a factor warning poor prognosis for the functional status of patients on the 90th day after onset (Lasek-Bal et al., 2015). In addition, when exogenous BDNF is given immediately (within hours) after CIS, it can act as a neuroprotective agent. Surprisingly, when it is given several days after ischemic injury, it can promote axons protruding budding and synapses formation (Nagahara and Tuszynski, 2011). However, BDNF cannot penetrate the undamaged blood-brain barrier, which makes peripheral BDNF injection ineffective. What’s more, transcranial BDNF treatment makes the time of tissue distribution much too short. Therefore, the way of delivering BDNF need further research to achieve the optimal treatment (Cook et al., 2017).

## Conclusion

In this review, we introduced the physiological and pathological functions of BDNF as well as its potential treatment effect in CNS diseases including depression, Alzheimer’s disease, Parkinson’s disease, Huntington’s disease, multiple sclerosis, amyotrophic lateral sclerosis and cerebral ischemic stroke. We discussed the alteration of BDNF level and BDNF-based therapies in different disease models. Apart from the disorders above mentioned, the role of BDNF was also investigated in many other diseases such as diabetes and cancer (Eyileten et al., 2017; Guzel et al., 2021). Understanding the role of BDNF and its signaling as well as how they regulate repair or regrowth of CNS will be useful. Myriad compelling studies reveal that enhancing BDNF levels or restoring its receptor’s signaling cascades can improve the phenotype of animal models in different diseases. However, there are still many questions to be answered. Firstly, BDNF has a large molecular weight and is difficult to pass through the blood-brain barrier, so it cannot reach the CNS and play a role when intravenous administration. Intraventricular injection has high technical requirements for operators and is prone to cause huge damage, which leads to it difficult to popularize. Using BDNF agonists to increase the release of BDNF *in vivo* can solve this problem, but what agonists we should use is another problem. Recently, more and more researchers take their emphasis on BDNF-overexpressed stem cells transplantation in animal model, which might be a promising therapeutic strategy but it is on the try phase and further studies are needed. Secondly, at present, BDNF is still limited to animal experiments and cannot be applied in clinical practice on a large scale. Thirdly, there is no consensus on when and at what dose BDNF should be administered to specific diseases.

## Author contributions

Z-HW conceived and revised the manuscript. YL, FL, DQ, and HC found the associated documents. JHW, JBW, SS, CW, YW, SL, and DG helped to draw the figures. YL wrote the manuscript with input from all authors. All authors contributed to the article and approved the submitted version.
